# Practice of the New Integrated Molecular Diagnostics in Gliomas: Experiences and New Findings in a Single Chinese Center

**DOI:** 10.7150/jca.38603

**Published:** 2020-01-01

**Authors:** Wan-Ming Hu, Fang Wang, Shao-Yan Xi, Xiao Zhang, Jun-Peng Lai, Hui-Yu Wu, Li-Ling Liu, Ke Sai, Jing Zeng

**Affiliations:** 1 Department of Pathology, Sun Yat-sen University Cancer Center; State Key Laboratory of Oncology in South China; Collaborative Innovation Center for Cancer Medicine, Guangzhou, Guangdong, P. R. China;; 2 Department of Molecular Diagnostics, Sun Yat-sen University Cancer Center;; 3 Department of General, Sun Yat-sen University Cancer Center;; 4 Department of Neurosurgery, Sun Yat-sen University Cancer Center;

**Keywords:** IDH, ATRX, 1p/19q, TERT, MGMT, FISH, Sanger sequencing, glioma, glioblastoma

## Abstract

**Background:** The latest WHO classification of CNS tumors using the integrated phenotypic and molecular parameters (IDH, ATRX, 1p19q, TERT etc.) have reestablished the CNS tumors classification in addition to traditional histology. The establishment of glioma molecular typing can more accurately predict prognosis, better guide individualized treatment to improve survival.

**Methods:** The expression of IDH1, ATRX, PHH3, P53 and Ki67 was detected by IHC. Molecular status of IDH1/2 and TERT were analyzed using Sanger sequencing. MGMT was explored using methylation-specific PCR. 1p/19q codeletion status was firstly detected by FISH, then further confirmed by multiplex PCR-based next generation sequencing.

**Results:** The mutation frequency of IDH1 was 68.7% (79/115) in WHO II astrocytoma, and 82 cases (82/344, 23.8%) were “triple-negative glioma” in our cohort. Multivariate COX analysis revealed that only IDH, 1p/19q, TERT and MGMT were independent prognostic factors. Noteworthily, we found 7 cases of the new molecular phenotype presented as “IDH wildtype and 1p/19q codeletion”, not mentioned in the latest WHO guideline.

**Conclusion:** We detected the newly recommended markers in a large cohort of Chinese glioma patients. Our data demonstrated a relatively lower frequency of IDH mutations and a higher prevalence of triple-negative glioma in Chinese compared with American and European, indicating ethnic and geographical difference in some markers. In addition, the new molecular phenotype “IDH wildtype and 1p/19q codeletion” glioma deserved special focus. These findings suggest that further stratification of infiltrating gliomas is needed for different treatment strategy and precision medicine.

## Background

Glioma is the most common malignant and highly aggressive brain tumors, possessing the characteristics of infiltrating growth and easy recurrence. The treatment is very difficult and currently considered to be incurable. The total prognosis of gliomas was dismal, especially in glioblastoma multiforme's (GBMs). GBMs account for 30% of all brain and CNS (central nervous system) tumors and 80% of all malignant brain tumors 1. Although after several years of research, advancements in surgical techniques, and improvement of clinical care, GBMs still retains the poor prognosis of roughly 10 to 15 months. At the same time, different kinds of gliomas have a large heterogeneity. The survival ranges widely, from few months to several years. The imperfect classification of gliomas has restricted the development of new therapies for gliomas. Traditionally, the pathological diagnosis of glioma is almost entirely dependent on histomorphological features, and is classified into WHO grade I to IV. This tendency to subjective diagnosis leads to variability in outcomes between different pathologists and inability to accurately interpret prognosis 2. In 2010, Verhaak et al 3 published a landmark paper that puts forward the idea that glioblastoma have been divided into four subtypes base on the differentiated molecular characteristics. Four subtypes were namely classical, mesenchymal, proneural and neural. More recently, intensive research on the molecular genetic changes of glioma has led to the adoption of more and more molecular markers by WHO as diagnostic, prognostic and therapeutic indicators 4. The different subtypes of gliomas have been refurbished by the latest 2016 WHO criteria which provide the comprehensive diagnosis with phenotype and genotype feature compared with the traditional histologic classification. Using a lot of molecular parameters in addition to histology, and molecular testing of brain tumors becomes a crucial part of the diagnosis of glioma.

The purpose of this study was a retrospective analysis of 544 infiltrating glioma samples in our center. The expression of these markers in the population of gliomas in southern China was determined and compared with existing studies in the world to find out the differences and characteristics in order to further improve the diagnosis of glioma in the future, and lay the foundation for future targeted therapies.

## Methods

### Patients and Tissues

A cohort of 544 infiltrating glioma cases (WHO II-IV) were obtained from the Department of Pathology, Sun Yat-sen University Cancer Center, Guangzhou, China, between 1998 and 2016. Samples and data anonymization have been approved by Ethics Committee of SYSUCC. And the samples were obtained the informed consent of the patient. The malignant series consisted of 176 cases of WHO II (astrocytoma and oligodendroglioma), 159 cased of WHO III (anaplastic astrocytoma and oligodendroglioma) and 209 cases of WHO IV (glioblastoma). The ratio of male to female was 1.34:1. The median patient age at the time of primary surgery was 42 years. The median survival time was 29 months (range 0-188 months). All the tissues used for study were FFPE (formalin fixed and paraffin embedded) blocks.

### Immunohistochemistry (IHC)

Many of genetic discoveries can be interrogated with cost-effective immunohistochemical stains in gliomas. The most commonly used surrogates are IDH1, ATRX and P53. Immunohistochemistry studies were performed using standard techniques as described previously 5. IDH1-R132H (clone H09, 1:50; Dianova, Hamburg, Germany), ATRX (1:500; Sigma-Aldrich, St. Louis, MO, USA), and P53/PHH3/ Ki67(1:100; Dako, Carpinteria, CA) was detected using formalin-fixed, paraffin-embedded tumor tissue sections on an automated BenchMark Ultra (Ventana Medical systems, Roche, SW). Sections from known mutation-positive and immunoreactive tumors were used as positive controls. Negative controls consisted of sections incubated with Phosphate-buffered saline instead of the primary antibody.

### Interpretation standard of IDH1, ATRX, P53, Ki67 and PHH3

Most IDH positive cases were presented as a strong diffuse cytoplasmic immunoreaction of IDH1-R312H staining. However, a strong but mottled staining could be seen in very few cases, for these cases, a threshold of 30% of positive cytoplasmic staining was used for cut-off value 6 .

Nuclear ATRX loss was scored as ATRX mutation if tumor cell nuclei were unstained while nuclei of non-neoplastic cells such as endothelia, microglia, lymphocytes and reactive astrocytes were strongly positive 7. A threshold of 10% of strong positive tumor nuclei was used to assign immunopositivity for ATRX 8 and P53 9, which provide the most accurate prediction of mutation.

The Ki67 index was calculated as the average percentage of positive nuclei on the total number of nuclei in high power microscopic fields, at 400× magnification. The statistical score was using a two-grade scale, with low (0) and high (1), which was representative for “< 10%” and “≥ 10%”. Score 1 was defined as high Ki-67 expression 10.

The mitotic index (PHH3) was calculated for the exact positive nuclei (the number of mitotic figures) in high power microscopic fields, at 400× magnification. The statistical score was also low (0) and high (1), which was representative for “< 5” and “≥ 5” mitotic figures per 10 HPF.

All the IHC interpretation was performed by two certified neuropathologists in all cases.

### Molecular genetics

1. Fluorescent *in situ* hybridization (FISH) was performed to detect 1p and 19q deletion using Vysis FISH Probe Kit (Abbott Molecular, Illinois, USA). At least≥25% of counted nuclei presented one target signal and two reference signals will be considered as 1p or 19q deleted when 100 non-overlapping nuclei were counted.

2. Mutation status of IDH1/2 and TERT promoter was studied with Sanger sequencing. Hotspot codons IDH1 Arg132 (exon 4)/IDH2 Arg172 (exon 4) and the hotspot mutations of TERT promoter at positions C228T and C250T were detected on an ABI® 3130 Genetic Analyzer (Life Technologies, USA), as described in another research 11.

3. The promoter methylation status of the MGMT gene was assessed using methylation-specific PCR with the EZ DNA MethylationDirect kit (Zymo Research Corp., Orange, California, USA).

4. Multiplex PCR-Based Next generation sequencing. We used this method to confirm the true 1p/19q codeletion. Primers for several segments of chromosome 1p and 19q as well as barcoding adapter DNA oligos in the first and second enrichment separately and synthesized by Sangon Biotech (Sangon Biotech, Shanghai, China). Sequencing libraries were generated using multiplex PCR methods. Each reaction was cleaned once using Agencourt AMPure XP kit (Beckman, Indianapolis, USA) to remove unused primers, according to the manufacturer's specifications. The concentration of the barcoded PCR produced library was measured by Qubit 3.0(Thermo Fisher Scientific, MA, USA), and diluted amplicons were sequenced on the Ion Proton system (Thermo Fisher Scientific, MA, USA).

### Statistical methods

Associations between categorical variables were evaluated by use of 2 × 2 contingency tables and the Chi square (χ2) test. The association between parameters was assessed using Spearman correlation coefficient. Overall survival was calculated from the time of surgery until death or the last follow-up. Univariate survival analysis was performed using Kaplan-Meier curves and the log-rank test. Multivariate analyses were done, involving a Cox proportional hazards model for which values of p<0.05 were considered significant. Analyses were carried out using SPSS16.0 (Chicago, IL, USA).

## Results

### The IHC results of IDH1R132H, ATRX, P53, PHH3, Ki67 and the molecular status of IDH1/2 mutation, 1p/19q chromosomal deletion, MGMT promoter methylation, TERT promoter mutation: A relatively low rate of IDH mutation and a high proportion of triple-negative gliomas in Chinese

In our cohort, the total positive rate for each marker was showed as follows: IDH1R132H (43.3%), ATRX (58.4%), P53 (58.7%), PHH3≥5/10HPF (58.4%), Ki67≥10% (66.3%), 1p/19q codeletion (19.1%), TERT promoter mutation (36.2%) and MGMT promoter mutation (43.7%). Based on these results, we have come to the following findings:

1. The traditional WHO grade was associated with the patient's age, IDH1, ATRX, P53, PHH3, Ki67, 1p/19q status and TERTp mutation(p<0.001), not related to the patient's gender and MGMTp status.

2. IDH1R132H immunoreactivity in tumor cell components occurred in most WHO II astrocytoma, and sanger sequencing revealed 68.7% (79/115) of WHO II astrocytoma occurred IDH1/2 mutated in our cohorts. Meaningfully, the incidence is lower in both our cohort and CGGA dataset than other researches focused on European and American including TCGA dataset (Table 1).

3. Loss of nuclear ATRX expression was remarkably relevant with IDH1/2 mutations (p<0.001), on the contrary, ATRX loss was mutually exclusive with the total 1p/19q codeletion (p<0.001).

4. The frequency of ATRX loss was the highest in WHO II astrocytoma (57.1%), while PHH3 and Ki67 were both exhibited high expression rate in high grade gliomas (WHO III and IV).

5. When using the FISH method to detect the 1p/19q status, it was found that in addition to cases with 1p/19q codeletion, there were a few cases with 1p deletion alone or 19q alone, but these individual gene deletion cases had no prognostic value.

6. The TERT promoter mutation was the highest (44.9%) in the WHO IV glioblastoma, followed by WHO II oligodendroglioma with IDH mutation and 1p/19q codeletion.

7. In our study, unfortunately, the detection failure rate of MGMT promoter methylation was high. From the results obtained, it is known that the highest proportion of MGMT promoter methylation occurs in glioblastomas (51.4%). It is well known that it is associated with the efficacy of temozolomide chemotherapy (patients with MGMT mutations have a better prognosis).

8. In our cohort, 344 WHO II&III cases have full molecular information of IDH, 1p/19q and TERT, and a total of 23.8% (82/344) was triple-negative (IDH wildtype, TERT promoter wildtype and no 1p/19q codeletion). The results were similar to Ng, H K et al., for the frequency of triple-negative gliomas was higher in Chinese patients (17.4% in Chinese VS 7% in Caucasian) 27, 28.

**Figure 1:**
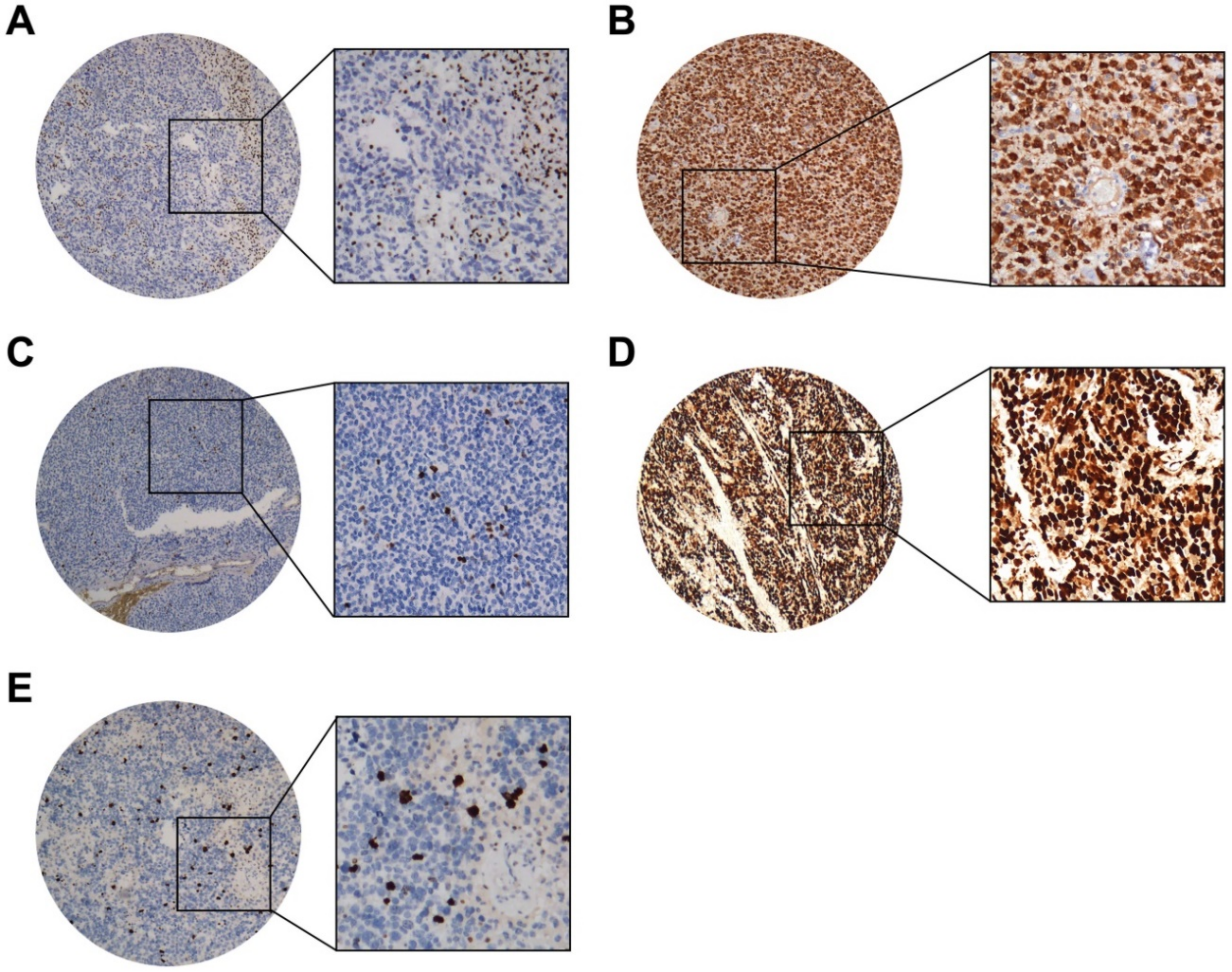
** Representative IHC staining cases of (A)** ATRX (nuclear loss expression while nuclei of non-neoplastic cells such as endothelia, microglia, lymphocytes and reactive astrocytes were strongly positive), **(B)** IDH1(strong diffuse cytoplasmic staining), **(C)** Ki67(Nuclear positive), **(D)** P53 (Nuclear positive), **(E)** PHH3 (Nuclear positive). (40X and 200X).

**Figure 2:**
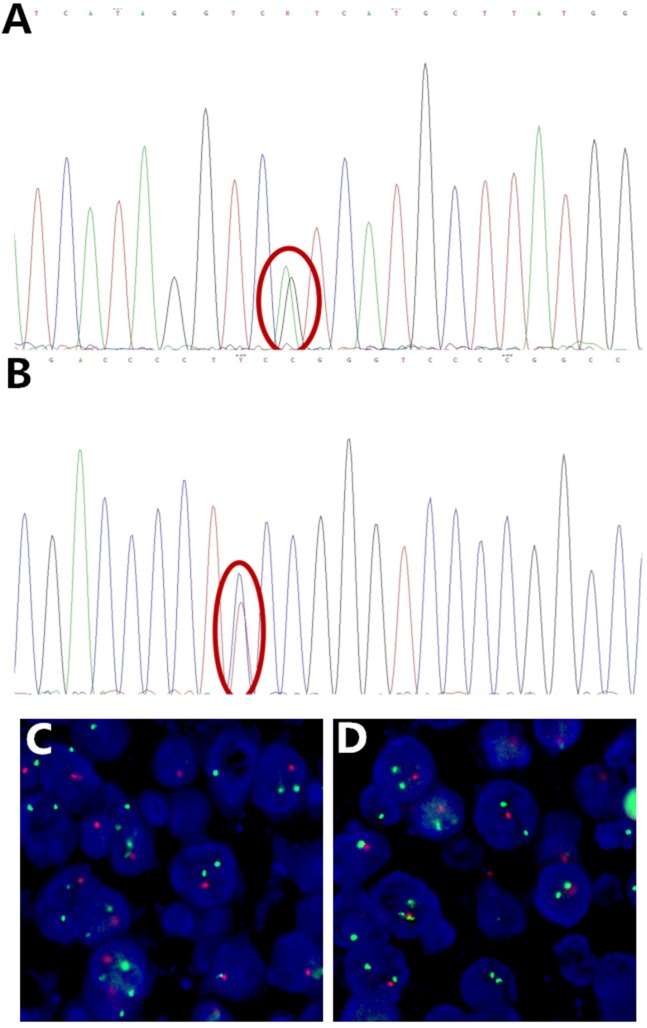
** Representative Sanger sequencing images of IDH mutation,** TERT promoter mutation and FISH image of 1p/19q deletion. **(A)** IDH1 R132H mutation (red circle), **(B)** TERT promoter C250T mutation (red circle), **(C)** 1p deletion, **(D)** 19q deletion, with 1 red and 2 green signals in scattered nuclei. Some signals are missing due to nuclear truncation and overlap.

The representative figures and detailed results were showed in Figure 1, Figure 2 and Table 2.

### Prognostic factors for infiltrating gliomas: IDH, 1p/19q, TERT and MGMT were independent prognostic factors

**Figure 3:**
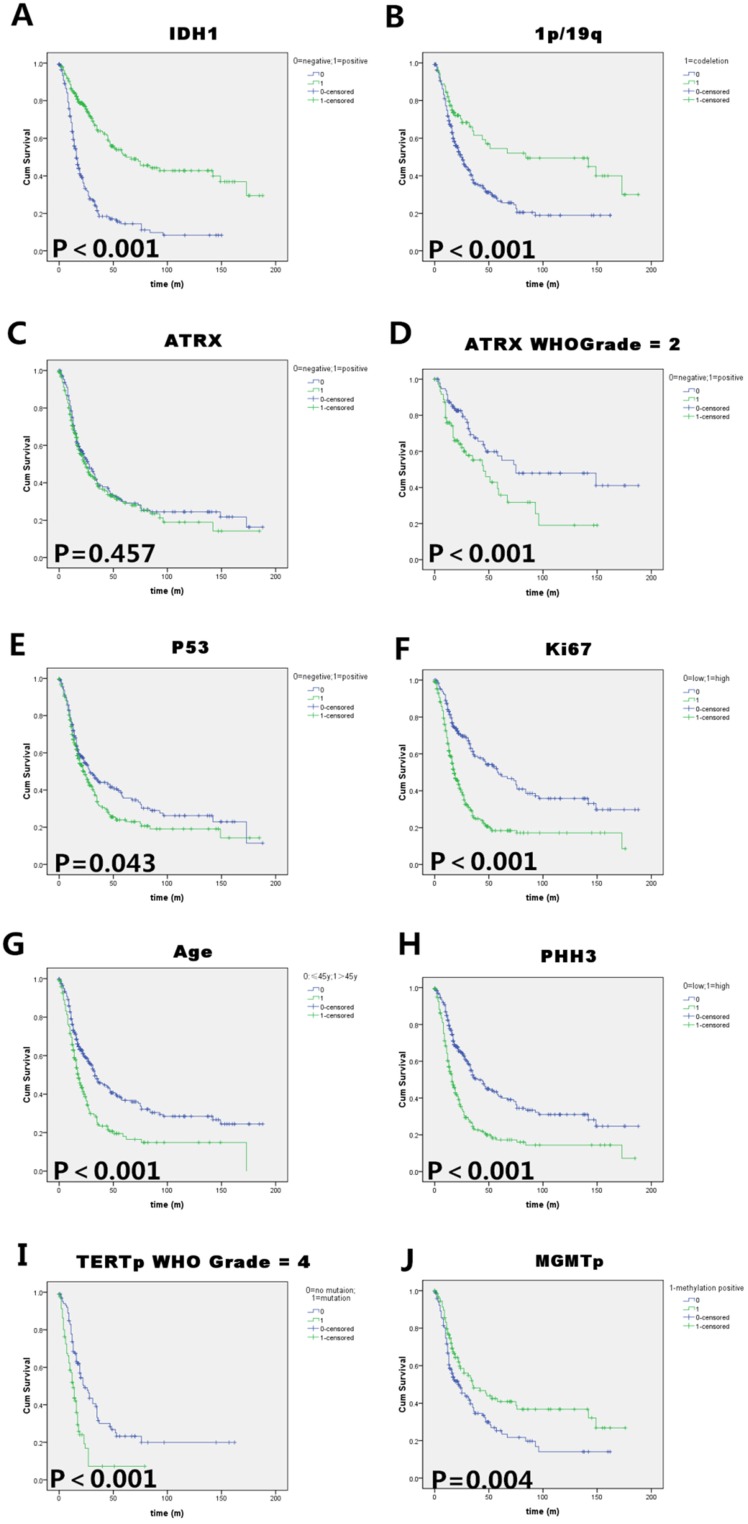
** Kaplan-Meier survival analysis of (A)** IDH1 mutation status, **(B)**1p/19q codeletion status, **(C)** ATRX immunoreactivity (negative=ATRX mutation), (D)ATRX status in WHO II astrocytoma patients, **(E)** P53 immunoreactivity, **(F)** Ki-67 labelling index (cut-off value=10%), **(G)** Patients' age at diagnosis (cut-off value=45 years), **(H)** PHH3(mitosis number/10HPF, cut-off value=5 ), **(I)** TERT promoter status in WHO IV glioblastoma patients and **(J)** MGMT promoter methylation status in all the infiltrating glioma patients. Log rank test p values are also showed for each parameter. IDH1 mutation, 1p/19q codeletion and MGMT promoter methylation were good prognostic indicators in all the infiltrating gliomas. Transitional markers such as high expression of P53/Ki67/PHH3 and old age exerted unfavorable prognosis. ATRX only have prognostic value in WHO grade II gliomas, and TERT promoter mutation only have prognostic value in WHO grade IV glioma (GBM) in our cohort.

We summarize the prognostic values of each marker in univariate analysis and the results of multivariate COX analysis in Tables 3, and Figure 3 shows the significant survival curves of each useful marker in glioma patients. In Kaplan-Meier univariate analysis, we observed that patients with IDH1-R132H positive tumors had a remarkably longer survival time than those with IDH1-R132H negative tumors (Median OS of IDH1-R132H positive=67 months, Median OS of IDH1-R132H negative=17 months; p<0.001).1p/19q codeletion patients had an extremely longer survival time (Median OS=84 months; p<0.001), but 1p deletion or 19q deletion alone didn't achieve statistical significance. ATRX loss was a favorable prognostic factor only in WHO II astrocytoma patients, but in all the glioma patients, it has no statistically significant (Median OS was 28 to 25, p=0.457). P53 mutation was a relatively unfavorable prognostic factor in our cohort (Median OS was 23 to 29, p=0.043). High PHH3/Ki-67 expression was associated with shorter OS in patients (Median OS was 16 to 45 and 19 to 59, respectively; both p<0.001). For the molecular status of TERT and MGMT promoter, the patients with TERTp wild type (especially in WHO IV/GBM) and MGMTp methylated had a better survival (Median OS of TERTp wild type=33 months, Median OS of TERTp mutated=22 months; p<0.001, Median OS of MGMTp methylated=35 months, Median OS of MGMTp methylated=23 months; p=0.004). Furthermore, the multivariate analysis showed that IDH (p<0.001), 1p/19q (p<0.001) TERT (p=0.006) and MGMT (p=0.002) were independent prognostic factors, for the PR were 0.262, 0.394, 1.727 and 0.57, respectively. Notably, in our study, the traditional indicators of malignancy, PHH3 and KI67, were not independent factors of prognosis.

### Correlation of IDH1/2 Mutation with IDH1 R132H IHC

544 cases were evaluated for the expression of the IDH1R132H mutant protein by IHC, of which 225 (41.4%) were positive and 319 (58.6%) were negative. Then, all the cases were sent to molecular detection, and the results were effective in 482 cases (88.6%). The frequencies of IDH1 mutations detected by the Sanger sequencing were 214 cases (44.4%), with IDH1 R132H mutation accounted for the vast majority of IDH1 mutation (92.9%, 199/214), followed by IDH1 R132C (4.2%, 9/214), R132S (1.4%, 3/214) and R132G (1.4%, 3/214), but we only found one IDH2 R172K mutation WHO grade II case in our cohort. Our data showed that IDH1R132H IHC results completely matched the IDH1 mutation status, demonstrating high sensitivity and consistency of the anti-IDH1R132H antibody (clone: H09) compared with molecular detection. Details were shown in Table 4.

### Conflicting molecular testing results with the phenotype presented as “IDH wild type and 1p/19q codeletion”

Among 544 infiltrating glioma cases in our cohort, we firstly found 20 cases were IDH wild type (Sanger sequencing) and 1p/19q codeletion (FISH). Although FISH is an approved and common method for 1p/19q molecular testing on routinely FFPE tissues, it was defective in some cases in the interpretation of results such as a critical value, only focal deletion, accompanied by polyploid deletion and may lead to false positive results. Based on this possibility, we sent all these 20 cases to retest the sample using Multiplex PCR-Based Next Generation Sequencing, and finally identified 7 cases of the new phenotype “IDH wild type and 1p/19q codeletion”. The age range is 25 to 75 years old. The average age is 46, mostly located in the frontal lobe. Notably, all the 7 cases were ATRX positive in IHC (Wild type), and 5/7(71.4%) cases have TERT promoter mutation (Table 5). It has no prognostic significance in our cohort due to the limited cases. To further confirm the results, we searched online CGGA database, and also found 4 cases with the same phenotype of “IDH wild type and 1p/19q codeletion”, and details was shown in Table 5 of case 11-14. Significantly, it should be brought to the forefront: although only accounted for a very small percentage, it really existed “IDH wild type and 1p/19q codeletion” oligodendroglioma and glioblastoma (GBM with oligodendrocyte components in the old version of WHO blue book) in practice.

## Discussion

The World Health Organization (WHO) classification of tumors of the CNS is a standard and commonly used diagnostic system for brain tumor classification. The grading system was initially employed based on the morphological appearance of tumor cells and the outcome of tumors. However, in recent years, classical histopathology classification no longer meets current clinical needs. More and more molecular markers have emerged. Crucial molecular markers such as mutations in IDH, ATRX and 1p/19q codeletion status are now central in the pathological diagnosis of glioma.

Isocitrate dehydrogenase (IDH) mutation is one of the most important molecular markers in glioma. The most common IDH1 mutation is a heterozygous missense mutation that results in the replacement of arginine by histidine in codon 132 enzyme active site 29. IDH1 mutation status has significant favorable prognostic value, which has been reported world widely, irrespective of any other markers, and our data also showed the same result. According to reports, IDH1 exhibits high frequency mutations (70-90%) in WHO II astrocytoma 23, 29, 30. However, our data shows that the incidence of IDH1 mutation is lower in Chinese than Caucasian, and the same results of IDH2. In our cohort, we only found one case (1/482, 0.2 %) carried IDH2 mutation (R172K). As reported in the literature, IDH2 mutations are rare, comprising only about 2.7-6.7 % of all IDH1/2 mutation cases 31-33. Consistent with our results, other data also show that the frequency of IDH2 mutations in Chinese patients seems to be lower than in Westerner. A study from West China Hospital only found three IDH2 mutation cases (3/207, 1.4 %) in the IDH mutation Chinese patients, for two cases of IDH2 R172K and one case of R172M mutation 13, the frequency is similar to another study of IDH2(R172K) in Chinese patients (3/203, 1.5 %) 34. Interestingly, in other diseases which carry IDH mutations such as hematological disorders, mutation rate was also lower in Chinese patients in contrast with the findings reported by North American and European populations 35. These statistics indicate that IDH mutations may have ethnic and geographical difference, and this phenomenon is noteworthy and need to be further researched.

ATRX, alpha-thalassemia/mental retardation, X-linked, was first discovered in pancreatic neuroendocrine tumors 36. It is able to incorporate histone variant H3.3 into heterochromatin, causing changes in telomere length and genomic instability 37, 38. Several researches have figured out the role of ATRX in the diagnosis of diffuse glioma, with a higher rate of ATRX mutations in diffuse astrocytoma 39. And Cai JQ et al. 14 reported that decreased expression of ATRX can cause inhibition of migration, promotion of apoptosis and reducing of proliferation in glioma cells. Some studies recommended ATRX status and IDH1-R132H initially for the top layer of “integrated decision” followed by histological classification, WHO grade, and other molecular information 40.

In 1994, Reifenberger et al. 41 first discovered the loss of heterozygosity (LOH) of chromosome arms 1p and 19q in a lot of oligodendrogliomas. Later, a lot of studies 42-45 demonstrated that the patient with 1p/19q codeletion had a higher sensitivity to chemotherapy and a better prognosis for survival. The FISH method has a higher sensitivity than the polymerase chain reaction (PCR) method when detecting the 1p/19q codeletion. Hence, FISH method has been widely used in detecting the deletion of chromosome 1p and 19q in gliomas. We firstly found 91 cases of 1p/19q codeletion in our cohort when we adopted FISH method. However, 20 cases presented as paradoxical results of IDH wildtype and 1p/19q codeletion. In consideration of conventional FISH probes hybridize to regions containing only 0.4% to 1.5 % of the corresponding chromosome arms, and other special circumstances would yield a number of “false-positive results” 46 such as a critical interpretational value, only focal deletion and polyploid deletion, we furtherly detected these 20 cases of conflicting molecular information to confirm the IDH status and 1p/19q status using the method of “Multiplex PCR-Based Next Generation Sequencing”, and finally identified 7 cases of peculiar phenotype presented as “IDH wild type and 1p/19q codeletion”. The false-positive results of 1p/19q codeletion may be due to interstitial deletions of probe hybridization or monomeric regions, rather than complete loss of 1p and 19q 46.

As to the three most important markers (IDH, ATRX and 1p19q), we found the same results as ISN-Haarlem consensus and the latest WHO CNS guildline, the loss of nuclear ATRX expression was significantly correlation with the IDH1/2 mutations status in grade II gliomas (101/124, 81.45%, P<0.001), and ATRX was seemed mutually exclusive with the total 1p/19q codeletion. Out of 91 cases of 1p/19q codeletion, only one case was ATRX negative. However, surprisingly, although we found the most 1p/19q codeletion gliomas were IDH mutated (92.31 %) in our cohort, there were still 7 cases with IDH wild type (7.7 %) after secondary detection. According to current opinion, such phenotype was not existed in glioma and many previous studies have suggested that 1p/19q codeletion tumors were all accompanied by IDH mutations 7, 47. Under the menu of latest WHO blue book, we can only put these conflicting cases into oligodendroglioma, NOS, which lacking IDH mutation. However, articles about this situation of conflicting molecular results were published recently. Mizoguchi et al. 48 reported 8 glioblastoma cases with 1p/19q codeletion and without IDH1/IDH2 mutation by microsatellite analysis. Clark et al. 49 adopting the FISH and microsatellite analysis also detected 28 cases containing 1p/19q codeletion in total 491 glioblastoma cases, accounting for 5.7 % (28/491), and only one case harbors the IDH1-R132H mutation. All these results show IDH wild type GBM can have 1p/19q codeletion as the same as our cohort. However, besides the two cases of GBM presented as the phenotype of “IDH wildtype and 1p/19q codeletion” in our cohort, there are still 5 cases of typical oligodendroglioma morphology in our cohort. As mentioned in Fuller, G N's study 46, 6 thorny cases of 1p/19q FISH-positive and IDH wild-type did not meet the diagnostic criteria for "oligodendroglioma, IDH mutations, and 1p/19q codeletion." Ho-Keung Ng et, al 50 also found 5 in 185 cases(2.7 %) presented as “IDH wildtype and 1p/19q codeletion” and did not warrant a diagnosis of “oligodendroglioma, IDH mutant and 1p/19q co-deleted.” Our results also confirmed the presence of 1p/19q co-deleted and IDH wildtype oligodendrogliomas as described in the above studies. Although the entity of “oligodendroglioma, IDH wildtype and 1p/19q codeleted” only accounts for a very small percentage, we argue that such entity should be added to the pathological diagnosis category, and further exploration of other novel molecular markers is needed for characterization besides ATRX and TERT. Notably, other alternative methods are needed to assess the 1p/19q status when the FISH results do not meet clinical expectations or morphological criteria.

The telomerase reverse transcriptase (TERT) is a catalytic subunit with reverse transcriptase activity in telomerase. Mutations in the presence of the TERT promoter region have been reported in numerous cancers. Among gliomas, oligodendroglioma and glioblastomas (GBMs) were the most frequently affected tumors 51-55. These mutations occurred in 2 hotspot positions (C228T and C250T). As to the prognostic significance of TERT, differences results depend on subtype of the gliomas. We found it was a poor prognostic marker in GBM, but no significance in all the infiltrating gliomas. Sanson, M et al. 56 also found that the TERT promoter mutation was a strong and independent worse prognostic marker in GBMs, and is not related to IDH status. However, Yang P et al. 57 found that the presence of TERT promoter mutation in WHO II and III gliomas means a good outcome, and it has been an independent factor relevant to a good prognosis in the IDH mutation (IDH-mut) subgroup. Because gliomas with concurrent IDH mutations and TERT promoter most possibly have a coexisting 1p/19q codeletion 22, it suggests that co-occurrence of mutations in TERT promoter and IDH plays a role in the genesis of oligodendroglioma and the favorable prognostic effect of the triple-positive tumors(IDH, 1p/19q and TERT) might be caused by 1p/19q codeletion which trump over the negative effects of TERT promoter mutation 58.

In China, there are a lot of relatively undeveloped regions which can't perform molecular testing. We hope we can establish a simple and economic testing process for the primary hospital. Based on our research, routine immunohistochemistry panel for primary hospitals include: IDH1(R132H), ATRX, P53, PHH3(mitotic figures marker) and Ki67(cell proliferation index marker). Since ATRX loss was mutually exclusive with the total 1p/19q codeletion, we can get a preliminary classification by the above package.

On the treatment of glioma according to the current classification, low grade gliomas with a poor prognosis, most cases were IDH wildtype, should receive more intensive treatment, especially in triple-negative gliomas. However, high grade gliomas with good prognosis factors, such as having 1p/19q codeletion, could appropriately use conservative management without radiation in some cases, rather than the typical treatment consisted of varying combinations of surgery, chemotherapy, and radiotherapy, which was recently, to some extent, confirmed by Sorge C et al. 59, for they found a WHO III anaplastic oligodendroglioma with IDH wildtype and 1p/19q codeletion in a children only underwent a near total resection and had a complete, durable response to temozolomide alone. Meanwhile, we also need to focus on triple-negative gliomas, for they account a relatively high proportion in our Chinese cohort. A portion of WHO Grade II or III gliomas in this group have an aggressive course and are associated with unfavorable survival and should be considered as WHO IV, which suggests the need for early adjuvant therapies.

The main limitation of the study is its retrospective nature, and there are few missing data. Its clinical significance and findings need to be demonstrated by other studies in the future. Nevertheless, it is the largest study up until now concerning the new integrated molecular diagnostics in southern China.

## Conclusions

We screened recommended markers in a large cohort of Chinese glioma patients. Our data also demonstrated the subgroups of diffuse gliomas defined by the newly recommended markers possess distinctive prognostic characteristics, especially IDH mutation. IDH1R132H antibody, with good consistency with the test of gene detection, could be initially used for diagnostic and prognostic considerations in the clinical setting. Furthermore, we revealed the frequency of IDH mutation is relatively lower in Chinese compared with American and European, especially in IDH2.

Our clinicopathological study showed a higher prevalence of triple-negative gliomas (IDH wildtype, 1p/19q no-codeletion and TERT promoter wildtype WHO II&III gliomas) in Chinese glioma patients.

Importantly, we discovered the new phenotype of “infiltrating glioma, IDH wild type and 1p/19q codeletion”, which is conducive to further explore other potential molecular phenotype and further refine the classification of gliomas, and lay the foundation for future targeted therapy and precision medicine.

## Supplementary Material

Supplementary figures.

## Figures and Tables

**Table 1:** Frequency of IDH1 Mutations in WHO II astrocytoma Patients.

Country	Total	IDH1	IDH1
		Mutated	Mutated rate
China (present study)	115	79	68.70%
China 12	50	29	58.00%
China 13	206	145	70.39%
China 14	125	86	68.80%
China 15	37	25	67.57%
China 16	417	309	74.10%
China 17	111	72	64.86%
Italy 18	87	66	75.86%
France 19	47	40	85.11%
Germany 20	71	54	76.06%
Germany 21	431	332	77.03%
America 22	233	203	87.12%
America 23	81	70	86.42%
America 24	75	64	85.33%
America 25	159	141	88.68%
CGGA 26	117	82	70.01%
TCGA	415	323	77.83%

Compared with Caucasians, Asians have a relatively low frequency of IDH mutations rate; CGGA=Chinese Glioma Genome Altas; TCGA=The Cancer Genome Atlas

**Table 2:** The detailed results of all the markers in our cohort. (grouped by the WHO grade)

Variables		Grade II		Grade III		Grade IV		Total	P Value	
**Median age (year)**		36		41		49				
**Age**	≥ 45 years	135	76.70%	100	62.89%	88	42.11%	323	<0.001*	
	< 45 years	41	23.30%	59	37.11%	121	57.89%	221		
	Missing	0		0		0				
**Gender**	Male	102	57.95%	91	57.23%	119	56.94%	312	0.983	
	Female	74	42.05%	68	42.77%	90	43.06%	232		
	Missing	0		0		0				
**IDH1**	Positive	116	67.84%	73	47.40%	36	17.82%	225	<0.001*	
	Negative	55	32.16%	81	52.60%	166	82.18%	302		
	Missing	5		5		7		17		
**ATRX**	Positive	73	42.94%	99	65.13%	120	60.30%	292	<0.001*	
	Negative	97	57.06%	53	34.87%	79	39.70%	229		
	Missing	6		7		10		23		
**P53**	Positive	82	48.24%	91	58.33%	144	69.90%	317	<0.001*	
	Negative	88	51.76%	65	41.67%	62	30.10%	215		
	Missing	6		3		3		12		
**PHH3**	High (≥ 5)	9	5.17%	136	86.62%	171	83.41%	316	<0.001*	
	low(< 5)	165	94.83%	21	13.38%	34	16.59%	220		
	Missing	2		2		4		8		
**Ki67**	High (≥ 10%)	46	26.29%	118	74.21%	205	98.09%	369	<0.001*	
	Low (< 10%)	129	73.71%	41	24.26%	4	1.91%	174		
	Missing	1		0		0		1		
**1p/19q**	Co-deleted	42	26.42%	43	31.16%	6	3.37%	91	<0.001*	
	1p deleted	4	2.52%	7	5.07%	12	6.74%	23		
	19q deleted	6	3.77%	8	5.80%	15	8.43%	29		
	Intact	107	67.30%	80	57.97%	145	81.46%	332		
	Missing	17		21		31		69		
**TERTp**	Mutated	39	24.38%	55	38.73%	84	44.92%	178	<0.001*	
	Wildtype	121	75.63%	87	61.27%	103	55.08%	311		
	Missing	16		17		22		55		
**MGMTp**	Methylated	35	37.23%	31	39.24%	62	51.24%	128	0.058	
	Wildtype	59	62.77%	48	60.76%	59	48.76%	166		
	Missing	82		80		88		250		

**Table 3:** Univariate and multivariate analyses of different prognostic variables in infiltrating glioma patients

Parameters	Univariate analysis		Multivariate analysis (COX)			
	Median OS	p	RR	95.0% CI for Exp(B)		P
	(month)	(Log rank)		Lower	Upper	
**Gender**		0.278	1.176	0.826	1.674	0.368
Male	24					
Female	27					
**Age**		<0.001*	0.962	0.668	1.385	0.835
≤ 45 years old	33					
> 45 years old	18					
**WHO Grade**		<0.001*	1.311	0.734	2.343	0.360
Low (II)	62					
High (III, IV)	18					
**IDH1**		<0.001*	**0.262**	**0.162**	**0.425**	<0.001*
Mutated	67					
Wildtype	17					
**ATRX**		0.457	0.963	0.67	1.384	0.839
Mutated	28					
Wildtype	25					
**P53**		0.043*	1.18	0.808	1.724	0.392
Mutated	23					
Wildtype	29					
**PHH3**		<0.001*	1.129	0.734	1.738	0.581
<5	45					
≥5	16					
**Ki67**		<0.001*	1.064	0.642	1.765	0.809
<10%	59					
≥10%	19					
**1p/19q**		<0.001*	**0.394**	**0.236**	**0.581**	<0.001*
Codeletion	84					
Intact	25					
**TERTp**		<0.001*	**1.727**	**1.172**	**2.544**	0.006*
Mutated	22					
Wildtype	33					
**MGMTp**		**0.004**	**0.57**	**0.397**	**0.819**	0.002*
Methylated	35					
Unmethylated	23					

**Table 4:** IDH1R132H IHC (544 cases) and sequencing analysis of IDH1/2 mutation results (482 cases)

WHO Grade	IDH1(R132H) IHC n/N (%)	Sequencing analysis	
		IDH1 mutation n/N (%)	IDH2 mutation n/N (%)
II	116/171(67.8)	108/158(68.4)	1/158(0.6)
III	73/152(48.0)	70/140(50.0)	0/140(0.0)
IV	36/199(18.1)	36/184(19.6)	0/184(0.0)

**Table 5:** Cases of phenotype “IDH wild type and 1p/19q codeletion”

No.	Gender	Age	Morphological Diagnosis	Location	ATRX	TERT	OS
1	Male	44	Anaplastic oligodendroglioma	Right frontal temporal lobe	wildtype	C228T	13m(Dead)
2	Female	51	Glioblastoma with oligodendrocyte components	Left frontal lobe	wildtype	C228T	97m(Alive)
3	Female	57	Oligodendroglioma	Left frontal lobe	wildtype	C228T	21m(Alive)
4	Female	52	Anaplastic oligodendroglioma	Right temporal occipital lobe	wildtype	Wildtype	2m(Dead)
5	Male	51	Oligodendroglioma	Right frontal temporal lobe	wildtype	C228T	16m(Alive)
6	Female	47	Glioblastoma	Right frontal lobe	wildtype	Wildtype	14m(Dead)
7	Female	34	Oligodendroglioma	Left frontal lobe	wildtype	C250T	12m(Alive)
8*	*Female*	*75*	*Anaplastic oligodendroglioma*	*Right frontal lobe*	*wildtype*	*Wildtype*	*13m(Alive)*
9*	*Male*	*25*	*Oligodendroglioma*	*Left frontal lobe*	*wildtype*	*C228T*	*12m(Alive)*
10*	*Male*	*26*	*Oligodendroglioma*	*Right frontal lobe*	*wildtype*	*C228T*	*10m(Alive)*
11**	*Male*	*45*	*Anaplastic oligodendroglioma*	*NA*	*wildtype*	*NA*	*2429D(Alive)*
12**	*Female*	*56*	*Glioblastoma*	*NA*	*wildtype*	*NA*	*607D(Dead)*
13**	*Female*	*46*	*Anaplastic oligoastrocytoma*	*NA*	*wildtype*	*NA*	*1016D(Alive)*
14**	*Male*	*24*	*Anaplastic astrocytoma*	*NA*	*wildtype*	*NA*	*181D(Dead)*

*After the establishment of this database, until the publication of this article, three similar cases with “IDH wild type and 1p/19q codeletion” were found in approximately 400 cases of glioma newly treated and diagnosed in our cancer center. **4 cases (CGGA_1281, CGGA_669, CGGA_D06 and CGGA_D58) of “IDH wild type and 1p/19q codeletion” were found in CGGA datasets among 325 Gliomas using RNA sequencing. M=month, D=day

## Abbreviations

WHO: World Health Organization
IDH: isocitrate dehydrogenase
ATRX: alpha-thalassemia/ mental retardation, X-linked
TERT: telomerase reverse transcriptase
MGMT: O6-methylguanine- DNA-methyltransferase
CGGA: Chinese Glioma Genome Atlas
TCGA: The Cancer Genome Atlas
