# Supplementary figures and images for: Practice of the New Integrated Molecular Diagnostics in Gliomas: Experiences and New Findings in a Single Chinese Center
Source: J Cancer. 2020 Jan 1;11(6):1371–82. doi: 10.7150/jca.38603 (PMC6995369; doi:10.7150/jca.38603)

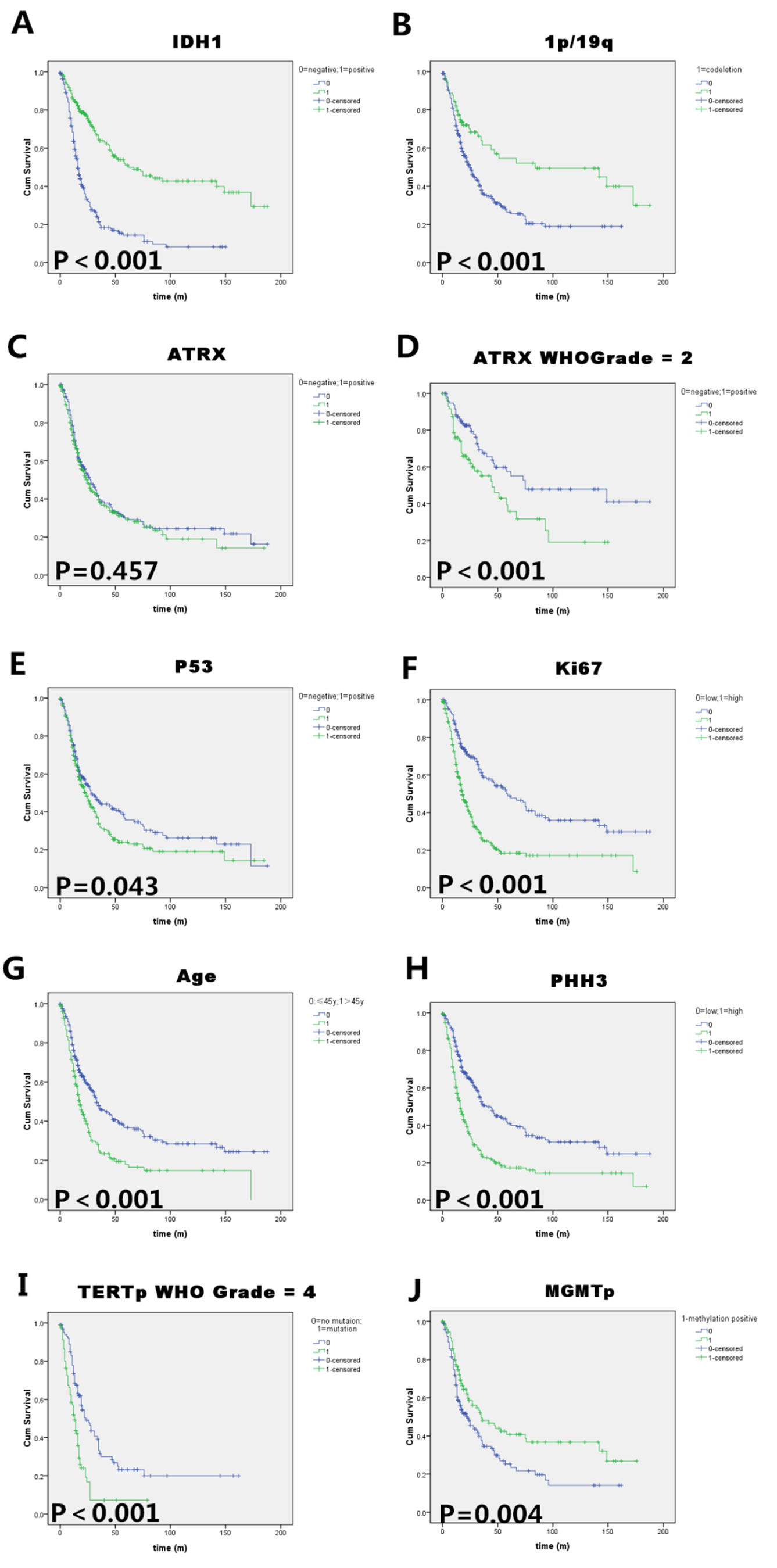

Supplement: Supplementary file 1 — Supplementary figures. [file jcav11p1371s1.zip › Figure 3.tif]

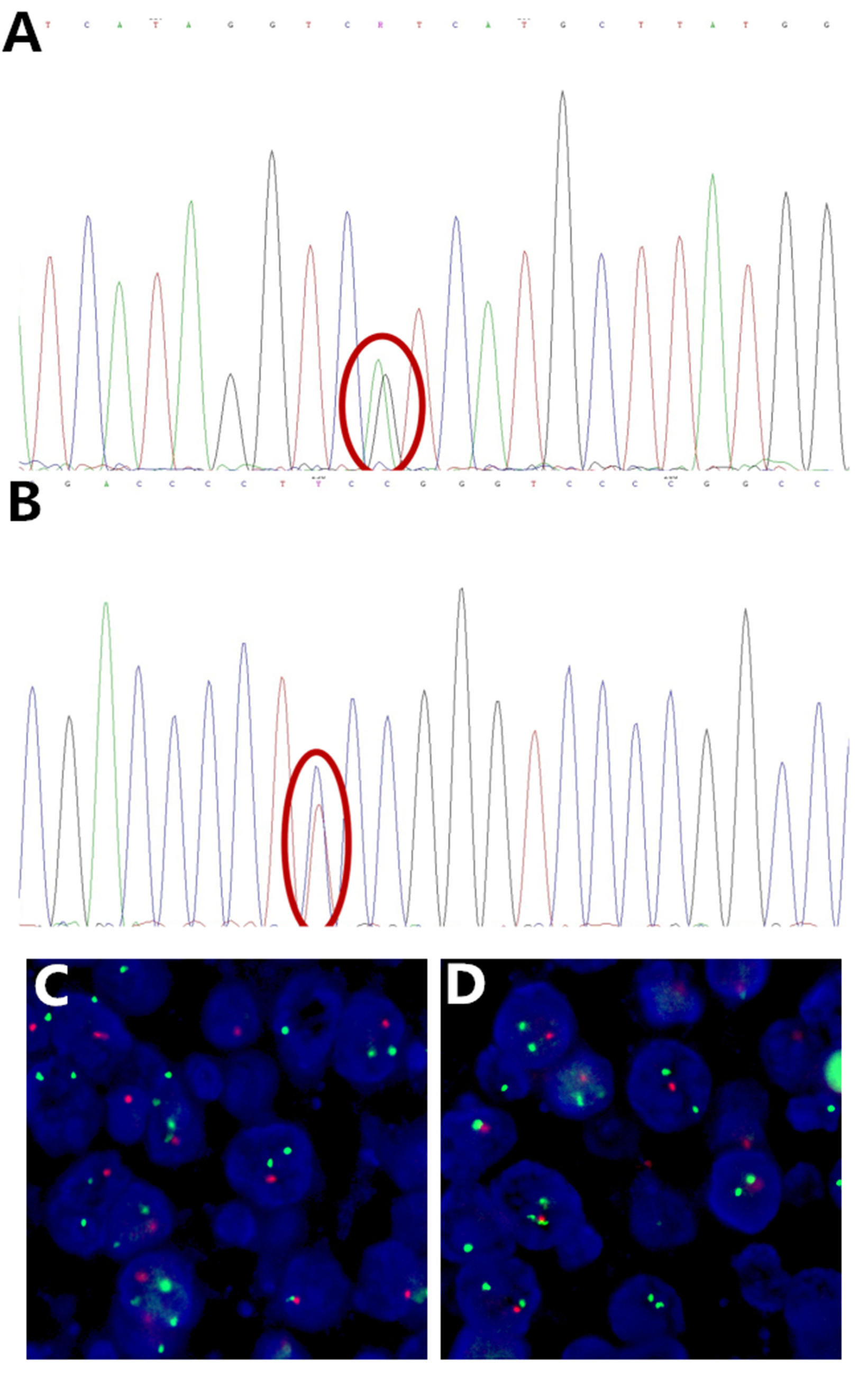

Supplement: Supplementary file 1 — Supplementary figures. [file jcav11p1371s1.zip › Figure 2.tif]

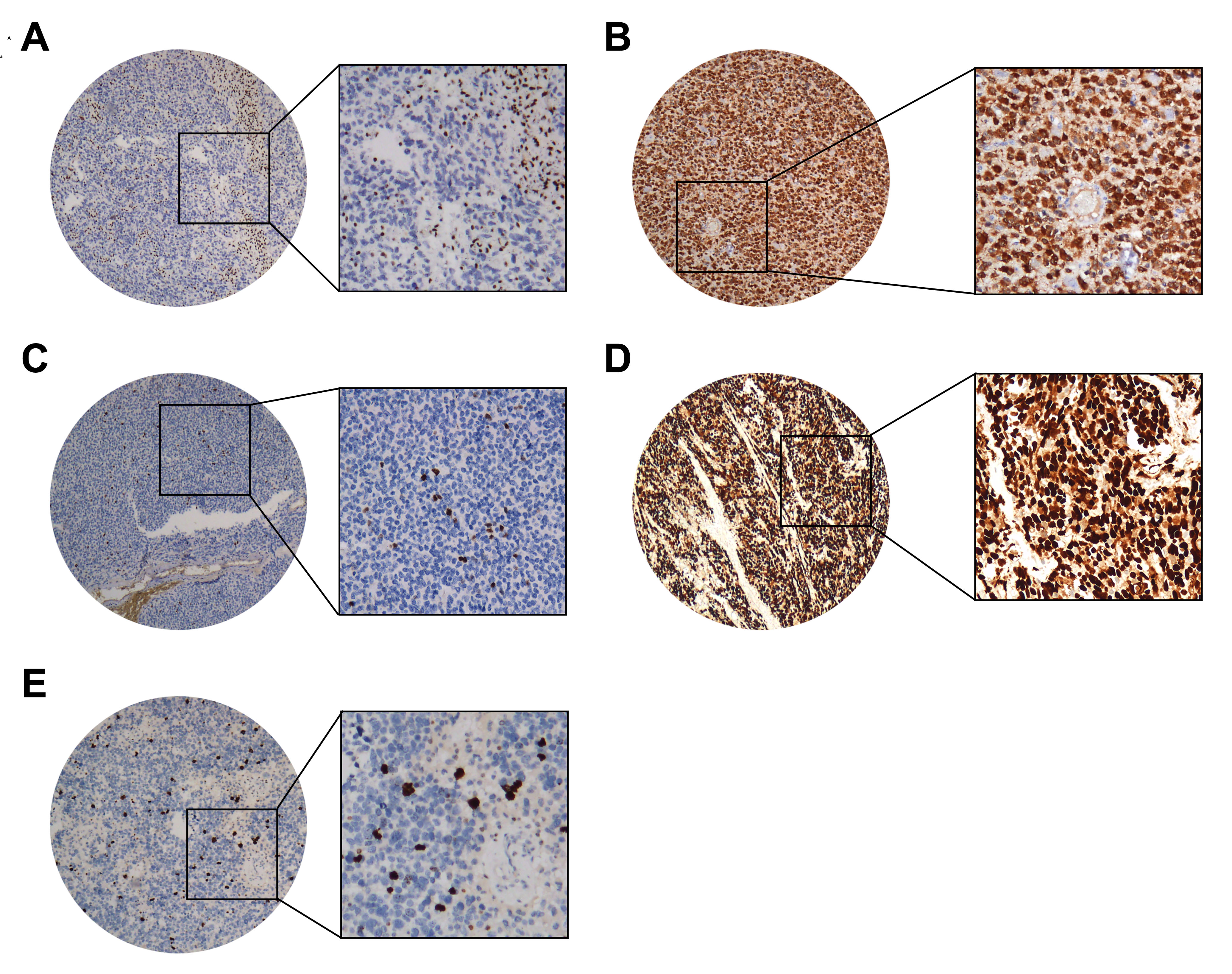

Supplement: Supplementary file 1 — Supplementary figures. [file jcav11p1371s1.zip › Figure 1.tif]
